# Immunotherapy in Metastatic Colorectal Cancer: Could the Latest Developments Hold the Key to Improving Patient Survival?

**DOI:** 10.3390/cancers12040889

**Published:** 2020-04-06

**Authors:** Emmanouil Damilakis, Dimitrios Mavroudis, Maria Sfakianaki, John Souglakos

**Affiliations:** 1 Department of Medical Oncology, School of Medicine, University of Crete, 71003 Heraklion, Greece; mavroudis@uoc.gr (D.M.); johnsougl@gmail.com (J.S.); 2 Laboratory of Translational Oncology, School of Medicine, University of Crete, 71003 Heraklion, Greece; mimasf19@gmail.com

**Keywords:** metastatic colorectal cancer, immunotherapy, immune checkpoint inhibitors, biomarkers

## Abstract

Immunotherapy has considerably increased the number of anticancer agents in many tumor types including metastatic colorectal cancer (mCRC). Anti-PD-1 (programmed death 1) and cytotoxic T-lymphocyte–associated antigen 4 (CTLA-4) immune checkpoint inhibitors (ICI) have been shown to benefit the mCRC patients with mismatch repair deficiency (dMMR) or high microsatellite instability (MSI-H). However, ICI is not effective in mismatch repair proficient (pMMR) colorectal tumors, which constitute a large population of patients. Several clinical trials evaluating the efficacy of immunotherapy combined with chemotherapy, radiation therapy, or other agents are currently ongoing to extend the benefit of immunotherapy to pMMR mCRC cases. In dMMR patients, MSI testing through immunohistochemistry and/or polymerase chain reaction can be used to identify patients that will benefit from immunotherapy. Next-generation sequencing has the ability to detect MSI-H using a low amount of nucleic acids and its application in clinical practice is currently being explored. Preliminary data suggest that radiomics is capable of discriminating MSI from microsatellite stable mCRC and may play a role as an imaging biomarker in the future. Tumor mutational burden, neoantigen burden, tumor-infiltrating lymphocytes, immunoscore, and gastrointestinal microbiome are promising biomarkers that require further investigation and validation.

## 1. Introduction

Colorectal cancer (CRC) is one of the most common cancer types along with breast, prostate, and lung cancer [1,2]. It also appears more frequently in males than females and, in both genders combined, displays the fourth highest mortality rate, after lung, breast, and prostate cancer [1]. Studies suggest that roughly 50% of CRC cases develop synchronous or metachronous distant metastases, typically in the liver or lung [3].

**Figure 1:**
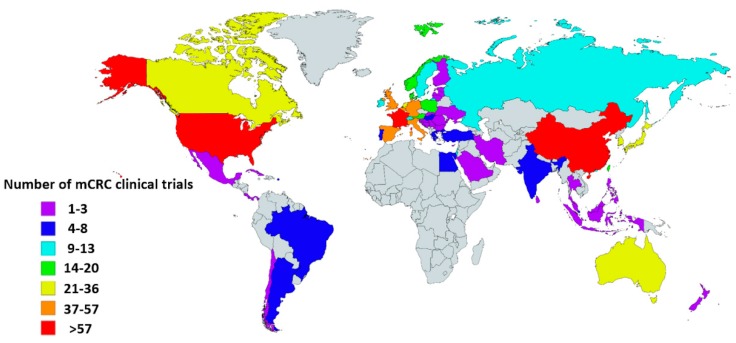
Global map showing the number of metastatic colorectal cancer (mCRC) clinical trials per country. Information was obtained from the USA-based registry ClinicalTrials.gov accessed on 12 February 2020. Eligible studies were ‘active, not recruiting’, ‘enrolling by invitation’, ‘recruiting’, and ‘not yet recruiting’ trials. All trials with ‘unknown status’ as well as ‘suspended’, ‘terminated’, ‘completed’, and ‘withdrawn’ studies were excluded. Information about these terms can be found at ClinicalTrials.gov. Gray indicates countries without clinical trials registered in ClinicalTrials.gov database. This map was created using an interactive map obtained from mapchart.net.

Currently, standard treatment for metastatic CRC (mCRC) includes chemotherapy, molecular targeted treatment, and surgery, though recently, rapid development has been made in the field of immunotherapy. The world map displayed in Figure 1 shows the number of mCRC clinical trials by country registered in ClinicalTrials.gov [4]. Many of these studies evaluating the efficacy of immunotherapy are currently ongoing in mCRC, either combined with or compared to chemotherapy.

As in all types of cancer, CRC is the result of mutations that accumulate over time. The three main molecular pathways that result in genomic instability are chromosomal instability (CIN), microsatellite instability (MSI), and CpG island methylator phenotype (CIMP) [5]. CIN is the most common type of genetic instability, present in up to 85% of CRC, impacting chromosome copy number and structure [6]. Patients with high microsatellite instability (MSI-H) amount to up to 15% of CRC cases. MSI-H is a result of DNA mismatch repair deficiency (dMMR), due to the inactivation of genes of proteins responsible for repairing errors, mainly occurring during replication. Such deficiency can be inherited, as is the case in hereditary nonpolyposis colon cancer (HNPCC), also known as Lynch syndrome, which accounts for roughly 3% of CRC cases [7]. Finally, in the case of CIMP, tumor suppressor and DNA repair genes can become silenced due to hypermethylation. CIMP can lead to acquired MSI-H, as opposed to hereditary MSI-H, and has been found to be associated with BRAF mutations.

Detecting MSI-H cases is crucial, as it determines the strategy for genetic analysis for Lynch syndrome. MSI-H is also a biomarker with an important prognostic and predictive value. Indeed, patients with early-stage CRC and MSI-H have better overall survival and lower chance of relapse [8]. However, MSI has not shown significant prognostic value in III or IV CRC [9]. Additionally, studies have shown that dMMR mCRC does not respond well to chemotherapy [10]. Instead, the high mutational burden related to MSI-H CRC results in tumors that express a plethora of neoantigens, making them highly immunogenic [11] and ideal targets for immunotherapy. Hu et al. [12] have suggested further stratifying MSI-H, which could enhance its prognostic and predictive value.

Cancer cells avoid detection and destruction by the immune system by utilizing several mechanisms [13]. Immunotherapy makes use of immune checkpoint inhibitors (ICI) to enhance immune response and overcome the resistance that tumors have developed. A lot of focus has been given on this promising field lately. The main aim of this review is to examine the various immunotherapy approaches in metastatic CRC and discuss biomarkers for the prediction of therapeutic effectiveness.

## 2. Immunotherapy for the Treatment of mCRC

### 2.1. Approved Immune Checkpoint Inhibitors in dMMR mCRC

The first study suggesting that MMR status predicts clinical response to immune checkpoint blockade was published in 2015 by Le et al. [14]. They conducted a phase 2 study to evaluate the clinical activity of pembrolizumab in 11 dMMR CRC patients, 21 mismatch repair proficient (pMMR) CRC patients, and 9 dMMR non-CRC patients. The results of this study showed that dMMR tumors are responsive to programmed cell death protein 1 (PD-1) blockade. Both CRC and non-CRC dMMR groups of patients experienced a high objective response rate (40% and 71%, respectively).

Immunotherapy is designed to amplify the patient’s immune system’s response to fight cancer cells. This is achieved by targeting checkpoint molecules, like cytotoxic T-lymphocyte–associated antigen 4 (CTLA-4), programmed cell death protein 1 (PD-1), and programmed cell death ligand 1 (PD-L1) proteins that inhibit immune response through negative feedback mechanisms. For MSI-H mCRC, only PD-1 and CTLA-4 inhibitors have been approved so far. CTLA-4 acts early in the immune response chain, by inhibiting T cell activation, while PD-1 acts in later stages, by causing T cell apoptosis [15]. Therefore, blocking the function of these proteins can lead to increased immune response.

Currently, Pembrolizumab, Nivolumab as well as the combination of Nivolumab and Ipilimumab have been approved by the Food and Drug Administration (FDA) for the treatment of MSI-H or dMMR metastatic CRC cases progressing after treatment with fluoropyrimidine, oxaliplatin, and irinotecan. An overview of selected clinical trials regarding these drugs can be found in Table 1 [16,17,18]. At the time of this writing, the European Medicine Agency (EMA) has not yet approved any of the aforementioned medicine for the treatment of MSI-H CRC. In fact, the application of Bristol-Myers Squibb to EMA’s Committee for Medicinal Products for Human Use (CHMP) for extending the use of Nivolumab to CRC patients was withdrawn, after CHMP deemed the results of the study as insufficient for approval [19].

#### 2.1.1. Pembrolizumab

Pembrolizumab is a PD-1 inhibitor for the treatment of unresectable or metastatic MSI-H or dMMR solid tumors that do not respond well to other forms of treatment [20]. It is available under the name KEYTRUDA. KEYTRUDA received accelerated approval by the FDA, based on a study that included 149 patients with MSI-H or dMMR cancer, 90 of which had CRC [21]. In this study, pembrolizumab displayed an objective response rate (ORR) of 39.6% (95% CI: 31.7, 47.9). Ahamandi et al. have studied the pharmacokinetic (PK) properties of pembrolizumab and found its half-life to be 27.3 days, exhibiting linear clearance of 0.22 L per day and a small volume of distribution [22].

#### 2.1.2. Nivolumab

Nivolumab, marketed under the name OPDIVO, is a PD-1 inhibitor for the treatment of MSI-H or dMMR CRC [23]. Like pembrolizumab, nivolumab also received accelerated approval by the FDA, based on a study on 74 MSI-H or dMMR CRC patients, which showed an ORR of 32% [24]. Bajaj et al. found the PK of nivolumab to be linear, exhibiting clearance that decreases over time, up to 25% of the original value, which may be related to improvement in cancer status [25].

#### 2.1.3. Nivolumab and Ipilimumab

Ipilimumab, sold under the trade name YERVOY, is a CTLA-4 inhibitor that was approved for the treatment of MSI-H or dMMR CRC, based on the results of the same study that Nivolumab monotherapy was approved [26]. In that study (Checkmate 142), 82 dMMR or MSI-H patients were treated with a combination of ipilimumab and nivolumab, followed by nivolumab monotherapy and had an ORR of 46% (95% CI: 35,58). A clinical update was published recently [27].

The combination of nivolumab and ipilimumab disables two different checkpoints that both downregulate immune response, thus resulting in better clinical response. Ipilimumab has a half-life of approximately 15 days and displays linear clearance that is steady over time, unlike nivolumab and pembrolizumab.

### 2.2. Adverse Events from Immune Checkpoint Inhibitors in dMMR mCRC

The use of ICI is associated with immune-related adverse events (irAE). Indeed, roughly 80% of patients receiving ICI treatment develop irAE within 3–4 months of therapy onset [28]. Immunotherapy’s adverse events are immunostimulatory, as opposed to those of chemotherapy, which are immunosuppressive. The most common irAE in mCRC include asthenia, diarrhea, rash, endocrinopathies, mainly hypophysitis and hypothyroidism, hepatitis, and pneumonitis [29]. In a clinical trial [14], 41 patients with pMMR and dMMR CRC or with dMMR non-CRC were treated with pembrolizumab. The study showed that grade 3 or 4 irAE occurred in 17% of cases. A similar percentage (20%) of grade 3–4 irAE was found in the CheckMate 142 clinical trial, which included 74 patients with dMMR mCRC treated with Nivolumab [17]. In a group of 119 dMMR mCRC patients, grade 3–4 irAE occurred in 32% of cases treated with a combination of nivolumab 3 mg/kg plus 1 mg/kg ipilimumab once every 3 weeks (4 doses) followed by nivolumab 3 mg/kg once every 2 weeks [18]. Preliminary results of a study on the efficacy and safety of nivolumab 3 mg/kg every 2 weeks and ipilimumab 1 mg/kg every 6 weeks in 45 patients with MSI-H/dMMR mCRC showed that grade 3–4 irAE occurred in 16% of patients. Seven percent of patients had to discontinue treatment due to an adverse event [27]. Less than 2% of irAE lead to death, with the most common causes being pneumonitis and hepatitis for PD-1 inhibitors and colitis and myocarditis for PD-1 + CTLA-4 inhibitor combination [29].

As biomarkers that predict toxicity have not yet been discovered, it is still impossible to accurately predict a patient’s risk profile prior to treatment. Therefore, it is of great importance to monitor patients for early detection of clinical symptoms that may alert health specialists to the presence of irAE. In case such symptoms are witnessed, guidelines should be followed for the appropriate course of action. Several such guidelines exist, created by associations such as the European Society for Medical Oncology (ESMO), the American Society of Clinical Oncology (ASCO), and the Society for Immunotherapy of Cancer (SITC) [30,31,32]. These guidelines rely on rating the individual patient’s irAE severity from grade 1 to grade 4, with patients suffering from grade 1 irAE typically continuing ICI treatment normally, simultaneously receiving symptomatic therapy. For grade 2 irAE, putting ICI treatment on hold is usually recommended, until the patient’s adverse event condition resolves. Grade 3 and grade 4 symptoms often require hospitalization and may even lead to permanent ICI treatment discontinuation. Corticosteroids are frequently recommended for symptomatic treatment, as is infliximab for higher grade irAE.

Careful patient monitoring and an interdisciplinary approach are essential to correctly evaluate the severity of the symptoms, as well as to rule out other potential causes that are not related to immunotherapy. However, due to the asymptomatic nature of several irAE making early detection difficult, as well as the fact that irAE might occur at any point during treatment or even after treatment discontinuation, effective and efficient monitoring is not always feasible. As a result, other methods may help reduce irAE frequency rate. One such method is using pre-drug formulations, where the antibody becomes active only after reaching the tumor site. Promising results have already been shown in the case of ipilimumab [33]. However, as previously mentioned, the discovery of biomarkers for accurate irAE risk prediction is of utmost importance and would greatly improve patients’ quality of life and survival.

### 2.3. Immune Checkpoint Inhibitors in pMMR mCRC

Current ICI are not effective in pMMR mCRC patients. The clinical trial by Le et al. on the activity of pembrolizumab [14] included not only patients with dMMR but also 21 pMMR patients with colorectal adenocarcinomas. Unlike dMMR, pMMR CRC patients did not respond well to pembrolizumab. Specifically, the objective response rate related to immunotherapy was 0%, the median progression-free survival was only 2.2 months, and the median overall survival was 5.0 months. Similarly, very limited response was observed in patients with pMMR in the study of Overman et al. [18].

Expanding the efficacy of ICI to pMMR patients, which constitute the majority of mCRC cases, will offer new perspectives in the treatment of mCRC. Several novel strategies are currently being investigated in clinical trials with the aim to enhance the immunogenicity of mCRC through combined therapies. The potential of bispecific antibody therapy such as the CEA-TCB has been recently observed in patients with mCRC [34]. The combination of ICI and anti-angiogenic treatment (bevacizumab) with or without chemotherapy has not led to an increased ICI response in pMMR tumors [35]. Preliminary results show that the strategy of combining avelumab, an anti-PD-L1 agent, with chemotherapy (FOLFOX) and a monoclonal antibody directed to epidermal growth factor receptor (EGFR), cetuximab, in RAS and BRAF wild-type mCRC may lead to an increased response [36]. The combination of ICI with mitogen-activated protein kinase (MEK) inhibition and chemotherapy is another promising approach currently under investigation [4]. If preliminary findings are confirmed in larger studies, it could have a considerable impact on the practice of oncology for the treatment of mCRC.

Research focused on the interaction between radiation therapy and the immune system has revealed new mechanisms that can be exploited to show clinical efficacy in solid tumors and specifically in pMMR mCRC [37]. It is of interest that effects of radiotherapy have been observed not only inside but also outside the radiation field. This phenomenon has been considered to have an immune origin and is known as the abscopal effect [38,39,40]. A study involving patients with multiple melanoma metastases was carried out to examine the feasibility, toxicities, and maximum tolerated fraction of stereotactic radiotherapy combined with immunotherapy [41]. Research findings of that study suggest that radiation therapy and checkpoint blockade may have synergistic tumor growth inhibition. A clinical trial of 2 investigational drugs (durvalumab and tremelimumab) administered in combination with radiotherapy or ablation in patients with mCRC is ongoing [4]. More research is needed to effectively evaluate the synergistic effects of radiotherapy and immunotherapy in metastatic patients. If a clinical benefit is demonstrated, it will provide new perspectives in the management of mCRC.

**Figure 2:**
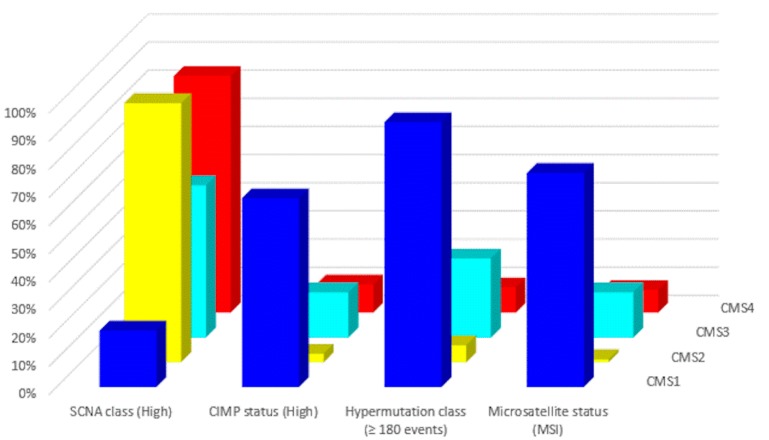
Molecular subtypes of CRC: Major molecular features (data from Reference [43]).

**Figure 3:**
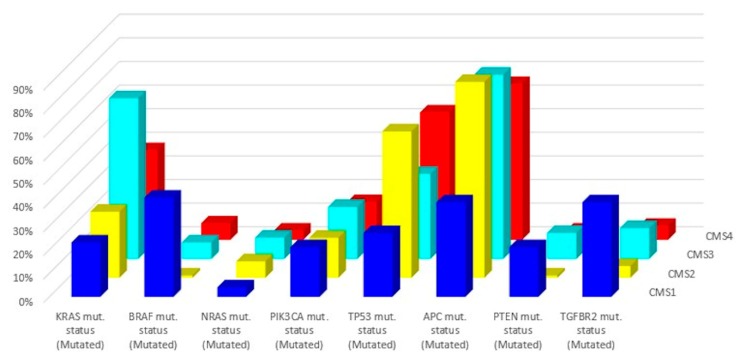
Related mutations for each molecular subtype (data from Reference [43]).

In 2015, a gene expression-based Consensus Molecular Subtype (CMS) classification system was created from an analysis of 4151 samples from stage II and III CRC patients [42]. Eighty-seven percent of the samples were assigned to a CMS (CMS1, CMS2, CMS3, CMS4), whereas 13% of cancers characterized by a mix of all subtypes remained unclassified. Key features of the 4 CMSs are presented in Table 2 and Figure 2 and Figure 3. The immune, inflammatory, angiogenic, and fibroblastic tumor microenvironment varies significantly between the 4 molecular subtypes [43]. CMS1 and CMS4 are ‘hot’ tumors characterized by strong immune infiltration. On the contrary, CMS2 and CMS3 are ‘cold’ tumors without immune activation. As Guinney et al. have pointed out [42], oncogene amplifications associated with CMS2 and the prominent metabolic activation of CMS3 tumors may be valuable tools for developing new therapeutic interventions in CRC patients. Several treatment strategies based on the 4 molecular subtypes of CRC have been proposed during the last years that could eventually overcome the absence of T cell infiltration and turn immunologically ‘cold’ tumors into ‘hot’ ones [44,45,46]. Research findings show that these approaches based on comprehensive analysis of CRC tumors have the potential to lead to customized treatments for individual patients, with improved clinical responses and fewer side effects.

### 2.4. Ongoing Clinical trials with ICIs in mCRC

Several drugs and drug combinations are currently under investigation in an attempt to identify new, effective immunotherapy options against mCRC [47]. Table 3 summarizes selected clinical trials studying several ICIs and combinational therapies that have not received approval from regulatory authorities yet.

## 3. Biomarkers

Currently, immunotherapy benefits only a fraction of cancer patients, and some of these patients are experiencing immune-related adverse events. Biomarkers are required to identify patients that will best respond to immunotherapy [48,49]. PD-1/PD-L1 expression is often used as a predictive biomarker of response to treatment in cancers other than CRC, such as non-small-cell lung cancer. However, studies on CRC patients have found no statistically significant difference in survival based on the level of PD-L1 expression [14,17,18]. Sections below provide an overview of biomarkers with relevance to response to immunotherapy in CRC.

### 3.1. DNA Mismatch Repair System Deficiency Testing

Methods based on immunohistochemistry (IHC), polymerase chain reaction (PCR), and next-generation sequencing (NGS) can be used to identify dMMR patients for immunotherapy. IHC and PCR methods to detect MSI have already been incorporated into clinical practice. The use of NGS to assess MSI provides advantages over PCR and its clinical use is currently being explored. Moreover, preliminary studies have shown that radiomics may serve as an imaging marker for discriminating MSI from microsatellite stable (MSS) CRC. 

#### 3.1.1. Immunohistochemistry (IHC)

Immunohistochemistry (IHC) tests tumor samples to examine loss of expression of MMR genes. Labeled monoclonal antibodies designed for the 4 major MMR proteins (MLH1, MSH2, MSH6, PMS2) are applied to tumor tissue samples to investigate protein expression. This procedure is based on visualization through staining the nucleus of cells. Lack of staining indicates no immunogenic response, as a result of no protein and gene expression. 

The MMR system includes 3 MutS homologs (MSH2, MSH3, and MSH6) and 4 MutL homologs (MLH1, MLH3, PMS1, and PMS2) that work in pairs to correct mistakes. Specifically, MSH2 forms heterodimers with MSH3 (MutSβ) and MSH6 (MutSα). MLH1 works with PMS2, PMS1, and MLH3 forming MutLα, MutLβ, and MutLγ, respectively. Consequently, loss of MSH2 function will result in loss of MSH3 staining, for example, because of MSH3 missing its partner. The reverse is not true, however, because MSH2 can still form the heterodimer with MSH6. IHC results indicate dMMR in case of a lack of one or more proteins.

IHC has several advantages that make it an appealing screening method. It is relatively inexpensive and can produce results fast, compared to other screening tests. Additionally, it is readily available, as it involves routine pathology lab procedures. Finally, it is important to note that IHC is capable of pinpointing specifically which gene is affected, allowing further mutation analysis.

However, there are also several drawbacks to IHC. A major disadvantage is that it requires an expert pathologist to perform because staining results can sometimes be ambiguous. Details about staining procedures can be found in the literature [50]. Furthermore, IHC cannot detect instability that does not result from one of the four major genes being inactive. The accuracy of the method is also impacted by the quality of the sample, as samples after chemotherapy or radiotherapy have been found to be of lower quality compared to those that have not received treatment [51]. Finally, it is important to note that sometimes there can be a gene product that is antigenic but not functional, giving a false negative. This is the case in missense mutations, for example, where proteins retain their antigenic properties, causing antibodies to bind to them, despite being non-functional. 

#### 3.1.2. Polymerase Chain Reaction (PCR)

Taking a more indirect approach, PCR identifies dMMR by examining microsatellite stability. Microsatellites are short sequences of DNA, one to six base pairs in length, usually repeated 5 to 50 times. They are prone to errors that typically impact their length, due to insertion or deletion of repeat units, compared to germline DNA. When the MMR system is deficient, such errors are not corrected, resulting in microsatellite instability, which can be detected using capillary electrophoresis.

In an attempt to establish standardization, the US National Cancer Institute developed the Bethesda Guidelines in 1996 [52], listing the criteria under which CRC samples should be tested for MSI, as well as recommending a marker panel to be used in laboratories, which included 2 mononucleotide markers (BAT 25, BAT 26) and 3 dinucleotide markers (D2S123, D3S456, and D17S250). If 2 or more markers were found to be unstable, the sample was categorized as MSI-H. In cases where no instability was observed, the sample was characterized as MSS. In the event of instability on one marker, the tumor was classified as MSI-L. However, MSI-L and MSS are usually grouped due to similar clinical behavior.

**Figure 4:**
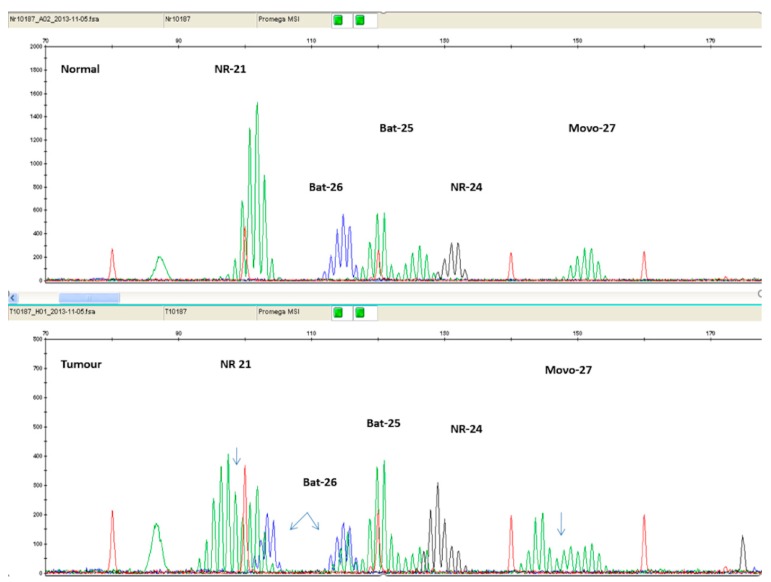
Electropherogram comparing normal tissue sample (top panel) with tumor tissue sample (bottom panel) of the same patient. The additional peaks present on the bottom panel are a result of MSI.

It has been found that mononucleotide markers have better sensitivity and specificity compared to dinucleotide markers [53]. Additionally, mononucleotide markers exhibit significantly lower polymorphism, compared to dinucleotide ones [54]. Therefore, panels consisting entirely of mononucleotide markers have been suggested. The current golden standard is considered to be the Microsatellite Instability Analysis Kit by Promega Corporation (Madison, WI, USA), which includes the markers BAT 25, BAT 26, NR 21, NR 24, and MONO-27. Figure 4 illustrates PCR results of an MSI-H patient, using the Promega Kit.

Since the PCR-based method evaluates the effectiveness of the MMR system as a whole, it is capable of identifying MSI that stems from mutations in any gene, as opposed to IHC’s limited 4 proteins. As a result, a mutation in PMS1, for example, would be identified through PCR, but not by IHC. Additionally, PCR produces results that are less obscure to interpret, leading to almost 100% reproducibility [55].

PCR has its limitations. An important disadvantage is that it is occasionally unable to detect mutations. For example, patients with a MSH6 mutation will not be classified as MSI-H. The most likely explanation is believed to be the partially redundant function of MSH6 and MSH3 proteins [56]. Moreover, PCR is less time-efficient and more expensive than IHC, which means that not all laboratories are capable of incorporating this screening test in their routine.

#### 3.1.3. Next-generation sequencing (NGS)

Next-generation sequencing (NGS) has been reported to be a very powerful approach for nucleic acid sequencing [57,58]. It permits parallel high-throughput sequencing of microsatellites and genes. NGS workflows include DNA extraction, DNA processing, library preparation, amplification, and sequencing. Details about clinical NGS technologies can be found elsewhere [59,60]. MSI status detection using NGS requires the development of computational methods. MSIsensor (https://github.com/ding-lab/msisensor) is an algorithm to detect replication slippage variants at microsatellite regions and differentiate them as somatic or germline [61]. Newer version based on machine learning, MSIsensor2, detects MSI using tumor only or ctDNA sequencing data. MANTIS (Microsatellite Analysis for Normal-Tumor InStability) detects MSI from paired-end binary alignment map (BAM) files (https://github.com/OSU-SRLab/MANTIS). To determine MSI, this program needs a tumor BAM and a matched normal BAM file [62]. The algorithm mSINGS (MSI by NGS) (https://bitbucket.org/uwlabmed/msings/src/master/) compares microsatellite markers to a population of MSI-negative samples [63]. MSI-ColonCore method determines MSI status by NGS read-count distribution. Published data [64] show that this approach has accurate performance exhibiting high sensitivity (97.9%) and specificity (100%). Kautto et al. [65] used data from 6 cancer types, including colon and rectal adenocarcinomas, to evaluate the performance of MSIsensor, mSINGS, and MANTIS. MANTIS demonstrated the highest accuracy (97.1%) at the threshold of 0.4, followed by mSINGS (96.0%) at a threshold of 0.1 and mSISensor (95.4%) at a threshold of 3.5%.

NGS technology has the ability to detect MSI in a very large number of microsatellite markers using a low amount of nucleic acids. This is of great importance for CRC studies since the material needed for analysis usually comes from biopsied tumors. Another advantage of NGS is the ability to determine the tumor mutation burden (TMB), which is a measure of somatic cancer mutation prevalence. However, standardization of NGS workflows is needed to improve quality and comparability. Moreover, validation of NGS with other methods such as Sanger sequencing is important to increase the accuracy of the results. To move from biomedical research to clinical practice, the development of laboratory quality management procedures for NGS testing is also an essential requirement. Ethical challenges, data management, and data security issues have to be addressed. The American Society of Clinical Oncology and the College of American Pathologists have published recommendations to establish standardized classification, interpretation, annotation, and reporting of sequence variants associated with cancer [66]. Moreover, the U.S. Food and Drug Administration (FDA) has recently published guidance to provide recommendations for designing, developing, and validating tests that use NGS technology [67].

#### 3.1.4. Radiomics approaches to predict MSI status in CRC

**Figure 5:**
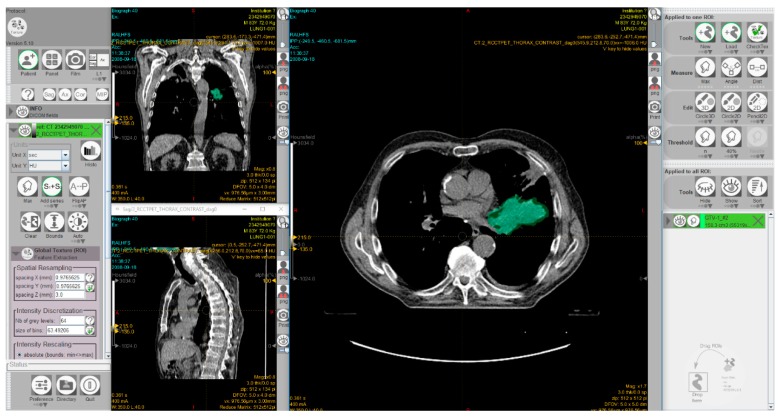
A screenshot of the graphical user interface of the freeware software LIFEx [70] for radiomic feature extraction. The interface shows computed tomography images of a patient with non-small cell lung cancer. The 3D volume of the gross tumor volume, manually delineated by a radiation oncologist, is shown in green. This exam was taken from the ‘NSCLC-Radiomics’ dataset [72,73] which is publicly accessible via ‘The Cancer Imaging Archive’ (TCIA; https://www.cancerimagingarchive.net).

Radiomics is the analysis of a high number of quantitative features, extracted from tumor regions, metastatic lesions, or normal tissues depicted in radiological images. Radiomics data combined with artificial intelligence algorithms can be used to identify predictive patterns of interest and support objective decisions for cancer diagnosis and treatment. However, there is a great demand for ‘big data’ sources. Substantial efforts have been devoted in recent years toward developing imaging biobanks that make image sets available for radiomics and other investigations. Thus, the National Cancer Institute (NCI) of the USA has created the open-source, open access ‘Cancer Imaging Archive’ to support research and educational activities related to radiomics and image processing [68]. This archive includes patient exams performed by various image modalities including cases treated with immunotherapy. Moreover, several software packages have been developed to allow radiomic feature extraction [69,70,71]. Figure 5 shows a screenshot of the graphical user interface of a software package used for radiomics. 

The first step of the radiomics procedure consists of the acquisition of the images and the delineation of regions of interest. Then, radiomic features are extracted and stored in a database. Information from other sources, such as clinical and molecular data, can also be included in the database. Data from all sources are subsequently combined and analyzed to develop diagnostic, therapeutic, prognostic, and predictive models. Among medical imaging modalities, computed tomography (CT) and positron emission tomography (PET)/CT are frequently used methods in ‘radiomics’ studies [74]. A very recent meta-analysis found poor diagnostic performance of 2-deoxy-2-[fluorine-18]fluoro-D-glucose (F-18 FDG) PET/CT for the prediction of KRAS mutation in colorectal cancer patients [75]. In the last years, interest has grown in radiomics using CT, which provides predictive information in several malignancies, including CRC. There has been compelling evidence that the heterogeneity of CT values within CRC liver metastases is associated with tumor grade and KRAS mutation [76]. A recent study showed that CT-based radiomics features can predict KRAS/NRAS/BRAF mutations in CRC [77]. Fan et al. [78] and Pernicka et al. [79] examined the potential association between MSI in CRC and CT radiomics features. The results of both studies showed that the prediction model based on both clinical and radiomics features achieved better predictive performance for MSI status in these patients than the model developed with either clinical data or radiomic features alone.

Dual-energy CT allows simultaneous image acquisition at two X-ray energies providing the opportunity to produce several CT datasets including material decomposition images. Iodine images show the concentration of iodine (mg/mL) within each voxel allowing the detection of small amounts of contrast material within a lesion and its distribution within tissues. The iodine concentration measured in iodine dual-energy CT images reflects the vascularization in a lesion. MSI tumors exhibit a higher angiogenic capacity than MSS [80] and this difference in angiogenesis may be demonstrated using dual-energy CT iodine images and radiomics analysis. Wu et al. [81] developed a model by incorporating multiple parameters to investigate the diagnostic accuracy of dual-energy CT for discriminating MSI from MSS CRC. The area under the curve (AUC) of the model provided relatively high diagnostic accuracy with an AUC value of 0.886, sensitivity 81.6%, and specificity of 81.6%. Another very recent study from the same institution examined the value of radiomics features extracted from dual-energy CT iodine images for predicting MSI in CRC [82]. The radiomics model achieved a good diagnostic performance showing the potential of radiomics analysis of iodine dual-energy CT images to predict MSI status in CRC patients.

Research results on the value of CT imaging for discriminating MSI from MSS CRC must be interpreted with caution as most findings are based on a limited number of patients from a single institution. Further multicentric studies are required using a larger sample size for such a technique to become implementable into everyday clinical practice. Moreover, radiomics features quantification may be sensitive to a number of technical parameters related to CT acquisition protocols, reconstruction techniques, and other factors [83]. Although research articles show that certain parameters or combinations of them derived from CT imaging may serve as an imaging marker for discriminating MSI from MSS CRC, radiomics features or any other models based on imaging data are not ready to replace the histopathological analysis and cannot be used in daily clinical practice yet. As in all emerging fields, more research needs to be conducted to validate the method.

### 3.2. Tumor Mutational Burden

Tumor mutational burden (TMB) is the total number of nonsynonymous, somatic mutations per coding area of a tumor genome expressed as the number of mutations per megabase. Tumor cells with high TMB are considered to be highly immunogenic because tumor mutations can form neoantigens that can be targeted by T cells [84,85,86]. Higher TMB increases the probability of a neoantigen that may be the target of T cells. TMB is estimated using DNA sequencing such as Whole Exome Sequencing (WES) or NGS. WES is expensive and time-consuming and, therefore, difficult to be implemented in everyday clinical practice. NGS platforms are both cost-effective and time-saving compared to WES and capable of providing a surrogate of TMB by profiling a small fraction of the coding exome.

Recent studies have shown that TMB has the potential to become a feasible predictor of response to immunotherapy across multiple cancer types [87,88]. Samstein et al. [87] have investigated the association between TMB and overall survival after treatment with ICI treatment in 1662 patients with stage IV or metastatic disease who had received at least one dose of ICI therapy. This study included mainly patients with non-small cell lung cancer (21.1%), melanoma (19.3%), and bladder cancer (12.9%). One hundred and ten patients (6.6%) had CRC. Higher TMB was associated with improved overall survival. It was also found that the TMB threshold differs across different cancers and, therefore, it is unlikely that one universal number will predict response to ICI across all histologies.

TMB is currently being studied as a potential predictive biomarker of response to PD-1 blockade therapy in CRC. Schrock et al. collected information for 22 mCRC patients with MSI-H tumors who were treated with PD-1 or PD-L1 inhibitors including 19 with pembrolizumab monotherapy [89]. MSI was confirmed by NGS. They found that patients with high TMB may respond effectively to ICI therapy. An important finding of that study was that the optimal threshold for TMB as a potential biomarker of response to ICI therapy in MSI-H CRC is from 37 to 41 mutations/Mb. Study findings suggest that a group of patients who do not respond to immunotherapy maybe those with MSI-H and TMB below the threshold of 37–41 mutations/Mb. These patients should be considered for chemotherapy rather than immunotherapy as their first treatment option. Further studies with a larger patient sample size are needed to validate the above results.

### 3.3. Neoantigen Burden

Several novel biomarkers for immune therapy have recently emerged that might receive approval by the regulatory agencies in the future. Neoantigen burden is the total number of neoantigens produced by somatic mutations of a tumor genome. A higher neoantigen burden is associated with improved immune therapy response [90,91,92,93]. The predictive value of neoantigen burden is still being evaluated and additional validation via clinical trials is needed. The lack of simple and cost-effective methods for neoantigen burden estimation is among the main issues that have to be addressed before this biomarker becomes a useful tool in clinical practice.

### 3.4. Tumor-Infiltrating Lymphocytes and Immunoscore

Evidence suggests that tumor progression is strongly dependent on the tumor microenvironment (TME). The presence of tumor-infiltrating lymphocytes (TIL) indicates an inflamed TME and is associated with MSI status and high neoantigen load. Smyrk et al. [94] proposed the quantification of TIL as a screening method for selecting CRC patients for MSI testing. They found that a TIL count of 5 yields a 93% sensitivity and 62% specificity for MSI-H status. Galon et al. have proposed the tool ‘immunoscore’ that is based on density, location, type, and functionality of immune cells within distinct tumor regions [95]. A recent study [96] showed that this tool can predict overall survival better than other parameters such as MSI status and provides a reliable estimate of the risk of recurrence in patients with stage I-III colon cancer. Future research can further develop such insights and assess the potential of immunoscore to predict CRC patients who will benefit from immune therapy.

### 3.5. Microbiome

The human gastrointestinal microbiome plays an important role in regulating immune status and, therefore, is another potential predictive biomarker for CRC immunotherapy [97]. Preclinical studies have found that the levels of specific microorganisms are considerably higher in tumors compared to those in nearby normal tissue. Data provided by Sivan et al. [98] support the hypothesis that a source of inter-subject heterogeneity concerning PD-1/PD-L1 therapeutic efficacy may be the composition of gastrointestinal microbes. Moreover, Vetizou et al. [99] found that the efficacy of the CTLA-4 blockade is influenced by the composition of *B. fragilis* and/or *B. thetaiotaomicron* and *Burkholderiales* microbiota. To use the gastrointestinal microbiome as a predictive biomarker in CRC, a better understanding of the functional role of microbiota is needed, as are a series of clinical studies translating preclinical results to approved biomarkers. 

## 4. Conclusions

Over the last years, there has been an accumulation of evidence that the two PD1-blocking antibodies, namely pembrolizumab and nivolumab, and one anti-CTLA4 antibody, i.e., ipilimumab, can considerably improve survival in many mCRC patients with MSI-H or dMMR. Multiple clinical trials are ongoing to evaluate other immunotherapy strategies for the treatment of dMMR/MSI-H mCRC. These studies have the potential to transform the way mCRC and other metastatic patients are treated and present an opportunity to establish immunotherapy as one of the most important pillars of treatment approaches in oncology.

Unfortunately, most pMMR/MSS mCRC patients, which represent the majority of mCRC cases, do not benefit from ICI alone. Ongoing clinical trials are currently investigating the possible role of various immunotherapy-based strategies in this group of patients. Bispecific antibody therapies, combinations of anti-PD-L1 agents with chemotherapy and monoclonal antibodies directed to the EGFR, as well as ICI combined with mitogen-activated protein kinase inhibition and chemotherapy are promising treatment approaches currently being evaluated in clinical trials. Moreover, studies assessing the efficacy of immunotherapy combined with radiation therapy are in progress. 

There is a need to identify the subset of mCRC patients who can benefit from ICI using biomarkers predictive of response. Currently, two methods, i.e., immunohistochemistry and PCR, are recommended for the detection of dMMR/MSI status in clinical practice. Studies have shown that NGS is a reliable method to assess MSI and its clinical use is currently being explored. Moreover, preliminary data suggest that radiomics are capable of discriminating MSI from MSS CRC and may play a role as imaging biomarkers in the future. TMB, neoantigen burden, TIL, immunoscore, and gastrointestinal microbiome are emerging, promising biomarkers that are under investigation. More research work is necessary for their clinical validation and approval by the regulatory authorities. 

## Figures and Tables

**Table 1:** Selected clinical trials of the Food and Drug Administration (FDA)-approved immune checkpoint inhibitors (ICI) for the treatment of high microsatellite instability (MSI-H)/mismatch repair deficiency (dMMR) patients.

Drug	Study	Phase	Target	Dose	Objective Response Rate (ORR)
Pembrolizumab	KEYNOTE 164	II	PD-1	200 mg/3 weeks	33%
Nivolumab	CheckMate 142	II	PD-1	3 mg/kg every 2 weeks	31.1%
Nivolumab + Ipilimumab	CheckMate 142	II	PD-1 and CTLA-4	First 4 doses: Nivolumab 3 mg/kg followed by Ipilimumab 1 mg/kg on the same day every 3 weeks Then: nivolumab 3 mg/kg every 2 weeks	55%

FDA. Food and Drug Administration; MSI-H, microsatellite instability high; dMMR, deficient mismatch repair; PD1, programmed death; CTLA-4, cytotoxic T-lymphocyte–associated antigen 4.

**Table 2:** The consensus molecular subtypes of colorectal cancer [42].

Subtype	CMS1	CMS2	CMS3	CMS4
**Taxonomy**	MSI Immune	Canonical	Metabolic	Mesenchymal
**Prevalence (%)**	14	37	13	23
**Age (years)**	69 (22–96)	66 (21–97)	67 (28–96)	64 (21–93)
**Location**	Proximal	Distal	Proximal or Distal	Distal

**Table 3:** Selected ongoing clinical trials in mCRC.

Clinicaltrials.gov Identifier	Drug(s)	Phase	Recruitment Status	Estimated Study Completion Date
NCT03150706	Avelumab	II	Recruiting	December 2021
NCT03555149	Regorafenib, Atezolizumab, Imprime PGG, Bevacizumab, Isatuximab, Selicrelumab, Idasanutlin, AB928	I/II	Recruiting	January 2022
NCT03435107	Durvalumab	II	Recruiting	May 2022
NCT02997228	Atezolizumab, Bevacizumab, Fluorouracil, Leucovorin, Leucovorin Calcium, Oxaliplatin	III	Recruiting	April 2022
NCT03982173	Tremelimumab Durvalumab,	II	Active, not recruiting	April 2023
NCT04262687	Capecitabine, Oxaliplatin, Bevacizumab, Pembrolizumab	II	Not yet recruiting	December 2023
NCT03711058	Copanlisib, Nivolumab	I/II	Recruiting	January 2022
NCT02834052	Pembrolizumab, Poly-ICLC	I/II	Recruiting	January 2021
NCT02851004	Napabucasin, Pembrolizumab	I/II	Active, not recruiting	April 2022
NCT03396926	Bevacizumab, Capecitabine, Pembrolizumab	II	Recruiting	January 2023
